# *H. pylori* Virulence Factors: Influence on Immune System and Pathology

**DOI:** 10.1155/2014/426309

**Published:** 2014-01-21

**Authors:** Behnam Kalali, Raquel Mejías-Luque, Anahita Javaheri, Markus Gerhard

**Affiliations:** Institut für Medizinische Mikrobiologie, Immunologie und Hygiene, Technische Universität München, 81675 Munich, Germany

## Abstract

*Helicobacter pylori* is the most widespread chronic bacterial agent in humans and is well recognized for its association with ulcer disease and gastric cancer, with both representing major global health and socioeconomic issues. Given the high level of adaptation and the coevolution of this bacterium with its human host, a thorough and multidirectional view of the specific microbiological characteristics of this infection as well as the host physiology is needed in order to develop novel means of prevention of therapy. This review aims to pinpoint some of these potentially important angles, which have to be considered mutually when studying *H. pylori*'s pathogenicity. The host's biological changes due to the virulence factors are a valuable pillar of *H. pylori* research as are the mechanisms by which bacteria provoke these changes. In this context, necessary adhesion molecules and significant virulence factors of *H. pylori* are discussed. Moreover, metabolism of the bacteria, one of the most important aspects for a better understanding of bacterial physiology and consequently possible therapeutic and prophylactic strategies, is addressed. On the other hand, we discuss the recent experimental proofs of the “hygiene hypothesis” in correlation with *Helicobacter*'s infection, which adds another aspect of complexity to this infection.

## 1. Introduction

*Helicobacter pylori* (*H. pylori*) is a helix shaped, microaerophilic, Gram-negative, flagellated bacteria. This bacterium is one of the most important human pathogens, infecting more than 50% of the human population. *H. pylori* and mankind have had an ancient relationship for at least 50,000 years [1]. Infection with *H. pylori* is usually acquired in early childhood and persists for life [2]. While over 80% of infected individuals are asymptomatic [3], the infection can lead to peptic ulcer, gastritis, and gastric cancer. Thus, being recognized as the principal agent leading to gastric cancer, WHO has classified *H. pylori* as a class I carcinogen. *H. pylori* uniquely colonizes the stomach where it induces inflammation and affects gastric physiology. There are well-characterized mechanisms of adaptation, which ancestral *H. pylori* have developed over the time. Through selection and coevolution, this bacterium established measures by which it actively and passively avoids the human immune response. Given the widespread prevalence of this infection, its socioeconomic impact, and the rising antibiotic resistance rates worldwide, novel means of treatment and prevention will be required. Therefore, it is essential to understand the unique metabolism capabilities, virulence factors as well as immune evasion mechanism of this bacterium, and its impact on human defense machinery.

The genome of this organism was fully sequenced in 1997 [4, 5], which facilitated and accelerated further studies on the biology, pathology, and immunology of *H. pylori *infection. Interestingly, its genome has a size of only one-third of *E-coli*'s genome [6], possibly reflecting the high degree of specialization of this bacterium. Beside *H. pylori*'s impressive tools which directly affect host cells and its binding molecules that facilitate anchoring of the bacterium to its host, the bacterium possesses metabolic factors which enable it to successfully alter the extreme environmental niche for its own benefit. Furthermore, there are comprehensive but mostly epidemiologic studies, describing a symbiotic relationship between man and *Helicobacter*. In the present review we will focus on bacterial factors involved in adhesion, pathogenesis, and inflammation as well as some key aspects of *H. pylori* metabolism, which will provide an insight into the biology of the bacterium and its symbiotic relationship with its human host.

## 2. *H. pylori*'s Adhesins

Adhesins are bacterial cell-surface proteins that enable bacterial adherence to cells. The adherence of pathogens to mucosal epithelial cells is the first step required for both colonization and pathogenesis. The adherence of *H. pylori* to the gastric mucosa is important for protection from mechanisms like acidic pH, mucus, and exfoliation [7]. *H. pylori* adhesins are considered as bacterial virulence factors and are involved in numerous processes during early and chronic phases of infection. They also contribute to the differential outcome in infected patients by triggering disease development. *H. pylori* adhesive factors belong to the largest outer membrane protein (OMP) family of the bacterium, namely, the Hop family. The Hop family contains the most well-known adhesins of *H. pylori* like BabA, SabA, AlpA/B, HopZ, and OipA.

### 2.1. BabA

The first identified and probably best characterized adhesin of *H. pylori* is a 78 KDa protein termed BabA (blood group antigen binding adhesion). BabA (HopS or OMP28) can bind to human Lewis^b^ (*α*-1, 3/4-difucosylated) and related terminal fucose residues on blood group antigens O (H antigen), A, and B on gastric epithelial cells [8, 9]. These initial studies were further substantiated in larger cohorts, which showed a coevolution and adaptation of this adherence factor with human blood group antigens serving as receptors [10–12].

At present *babA1* and *babA2*, which encode BabA, have been cloned [13], of which *babA2 is the *functionally active gene. It has been shown that the presence of *babA* gene correlates with the presence of *cagA* (cytotoxin-associated gene A) and *vacA* (vacuolating cytotoxin gene A), and the presence of all three genes increases the risk of gastritis, as well as the ulcer disease, gastric cancer, and MALT lymphoma [14]. On the molecular level, the BabA-mediated adherence to gastric epithelial cells is as an important pathogenic mechanism, which can influence the course of disease through aggravation of inflammatory responses in the stomach [12]. BabA/Lewis^b^ binding seems also involved in the induction of double-strand breaks of DNA and consequently DNA damage in host cells [15]. The immunological analysis of the inflammatory responses in the stomach revealed that BabA-positive strains colonize more densely and induce stronger IL-8 secretion in the mucosa compared to BabA-deficient strains [16]. Gerbils infected with BabA^+^ *H. pylori* strains showed higher levels of mucosal injury compared to BabA low-expressing or none-expressing strains [17]. BabA mediated binding of *H. pylori* to Le^b^ can trigger *cag*PAI-dependent host cell signaling and consecutive production of proinflammatory cytokines [18]. Interestingly studies in rhesus monkeys [19] and Mongolian gerbils [17] have shown that BabA expression is lost during longer course of infection, possibly because other adherence mechanisms take over. This could explain that changes in outer membrane protein expression may play a substantial role in *H. pylori* adaption to host gastric epithelium for promoting optimal adherence during chronic infection.

### 2.2. SabA

The sialic acid-binding adhesin HopP or OMP17 is a 70 kDa adhesin of *H. pylori* which binds to sialyl-dimeric-Lewis x (Le^x^) [20]. After initial colonization mediated by BabA, *H. pylori* infection leads to upregulation of Le^x^ expression, enabling SabA mediated binding. Interestingly, eradication of *H. pylori* decreases the expression level [21]. Moreover the adherence of *H. pylori* to extracellular matrix protein laminin is mediated by SabA [22].

The SabA adhesin can further bind the sialylated carbohydrates on granulocytes and induce an oxidative burst in these cells [23]. Moreover SabA binds to the sialylated structures expressed on erythrocytes and leads to hemagglutination [10]. The colonization density of *H. pylori* in patients lacking Le^b^ was maintained due to the SabA. Thus, in patients with weak or no Le^b^ expression, Le^x^ expression on the gastric epithelium plays a compensatory role in the maintenance of *H. pylori *colonization. [24].

### 2.3. AlpA/B

The highly homologous genes *alpa* and *alpb* encode the adherence associated lipoproteins AlpA (HopC or OMP20) and AlpB (HopB or OMP21) [4, 25]. The coproduced AlpA and AlpB proteins are involved in adhering to gastric tissue [26, 27]. Both proteins can bind to mouse laminin *in vitro* [28] and can induce the induction of IL-6 and IL-8 in gastric cell lines [29]. The absence of AlpA or AlpB not only reduced the bacterial load in the stomach in a guinea pig and gerbil model *of H. pylori* infection [30, 31] but also led to lower bacterial colonization in C57BL/6 mice [29]. At present no host receptor has been detected for either of these adhesins.

### 2.4. HopZ

Immunofluorescence studies have shown the presence of HopZ (74 kDa) on *H. pylori* cells. Furthermore, HopZ appears to mediate adherence to gastric epithelial cell lines as bacterial binding is significantly reduced in HopZ knock-out strains [32]. The exact function of HopZ is however still unclear. In a guinea pig model of *H. pylori* infection, the HopZ mutant strains did not affect the stomach colonization [31]. Conversely, HopZ inactivation reduced the ability of *H. pylori* to survive in the stomach in a transgenic mouse strain but not in the wild type controls in a model of chronic atrophic gastritis [33]. The host receptor for HopZ is as yet unknown.

### 2.5. OipA

The outer inflammatory protein A (HopH or OMP13) is a 35 kDa proinflammatory protein. The exact role of OipA is still not clear. While OipA was able to increase IL-8 secretion from gastric epithelial cell lines [34] and its combined function with cag PAI (the cag pathogenicity island) induced inflammation through phosphorylation of different signaling pathways [35–38], the mutant OipA strain could not alter *in vitro* IL-8 secretion from gastric cell lines [39], and inflammation in gerbils infected with OipA mutant strains was not attenuated [40]. The functional OipA expression of *H. pylori* is associated with duodenal ulcers and gastric cancer [40–42]. At present no host receptor for OipA has been identified.

## 3. *H. pylori* Virulence Factors Involved in Gastric Inflammation

The chronic inflammation elicited by *H. pylori* in the gastric mucosa plays a major role in the development of gastric cancer. Several bacterial virulence factors contribute to the inflammatory response towards *H. pylori* either by altering host-signaling pathways important to maintain tissue homeostasis in epithelial cells or by differentially stimulating innate immune cells. Of those, the cag pathogenicity island (PAI), CagA, and VacA are the best characterized. However, other bacterial determinants as *γ*-glutamyltranspeptidase (gGT), the duodenal ulcer-promoting gene (dupA), or peptidoglycan have been also shown to be important inducers of gastric inflammation.

### 3.1. CagPAI

Virulence strains of *H. pylori* possess the *cag*PAI. This 40 kb region contains 31 potential coding regions [43], which encode for the different components of a type IV secretion system (T4SS). Some of those components are essential for CagA translocation such as CagT [44] while others additionally play an important role in the host's inflammatory response. For instance, DNA recombination in CagY was found to alter the function of the T4SS and proposed to modulate the host immune response to promote bacterial persistence [45], while CagL induces inflammation by interacting with host integrins and inducing IL-8 secretion in a CagA translocation and NOD1-independent manner [46].

After assembly of the T4SS and pilus formation, CagA is translocated into host cells where it can undergo phosphorylation at EPIYA sites [47] by two types of kinases: SRC and ABL. SRC kinases mediate the initial phosphorylation of CagA, preferentially at EPIYA-C (and EPIYA-D) motifs, while ABL kinases phosphorylate any EPIYA site later during the course of infection [48]. Phosphorylated and nonphosphorylated CagA can interact with several host proteins and thus alter host cell signaling, playing a crucial role in *H. pylori*-induced inflammation. Several studies indicate that CagA can directly activate NF-*κ*B and induce the release of IL-8 [49, 50]. Moreover, NF-*κ*B activation and inflammation was significantly enhanced in the gastric mucosa of Mongolian gerbils infected with *H. pylori* CagA proficient bacteria. However, other studies suggest that activation of NF-*κ*B and IL-8 expression are dependent on the T4SS but CagA independent at early time points [51]. Nevertheless, while direct activation of NF-*κ*B and IL-8 upregulation remains controversial, it is clear that the presence of *cag*PAI drives the proinflammatory response of gastric epithelial cells. CagA is not only injected into gastric epithelial cells, but it can be also injected into B lymphoid cells [52] and murine and human dendritic cells (DCs) [53, 54]. Interestingly, CagA translocation into DCs suppresses host immune response by reducing the secretion of proinflammatory cytokines as IL-12p40 and enhancing the expression of the suppressive cytokine IL-10 [54], indicating a dual pro- and anti-inflammatory role for CagA during *H. pylori* infection dependent on the cellular context.

In addition to CagA, peptidoglycan can also be delivered into host cells through the T4SS and outer membrane vesicles [55]. Recognition of peptidoglycan by NOD1 induces the production of proinflammatory cytokines MIP-2, *β*-defensins, and IL-8 through activation of NF-*κ*B, p38, and Erk signaling in the host cells [56, 57]. Furthermore, activation of NOD1 by peptidoglycan regulates the production of type I interferon, which can affect Th1 cell differentiation [58]. Modifications in its structure seem to be essential for dampening host immune detection and contribute to bacterial persistence [59, 60]. Moreover, reduced mucosal cytokine response was detected in NOD1 deficient mice infected with *cag*PAI positive *H. pylori* strains [56], indicating that peptidoglycan-NOD1 signaling is important in the immune response towards *H. pylori*.

### 3.2. VacA

All *H. pylori* strains carry the *vacA *gene, which codes for the secreted pore-forming protein VacA. Expression levels, cell type specific toxicity, and disease severity are linked to sequence variation in different domains of VacA [61]. VacA is secreted by the bacterium via a type V autotransport secretion system and enters the host cells by endocytosis. Once internalized, VacA accumulates inside different cellular compartment and induces apoptosis [62]. In addition, VacA disrupts epithelial cell tight connections and is distributed in the lamina propria where it encounters T cells recruited to the sites of infection. As a result T cell proliferation and effector functions are inhibited, allowing persistence of the bacterium [63]. VacA has also been reported to have an indirect effect on T cells; the mechanisms are as yet unknown. VacA can induce DC tolerance and regulatory T cell induction; however this effect has not been yet documented in human cells [64]. Although VacA influences the host inflammatory response mainly by suppressing T cell activation, the toxin also induces a proinflammatory effect on T cells which is mediated by activation of NF-*κ*B and leads to upregulation of IL-8 [65]. Additionally, disruption of autophagy elicited by VacA is another mechanism by which it can cause gastric inflammation [66].

### 3.3. gGT

gGT is constitutively expressed by all *H. pylori* strains and gGT presence was shown to be essential for the establishment of the infection in mice [67]. It was shown that a *H. pylori* secreted-low molecular weight protein suppressed T cell proliferation [68]. Later studies identified this inhibitory factor as gGT and showed that disruption of the Ras signaling pathway was the molecular mechanism employed by gGT to induce T cell cycle arrest [69]. More recent data in murine models of infection as well as our own unpublished results in human dendritic cells indicate that gGT contributes to DC tolerization, skewing the T cell response towards a regulatory phenotype [64]. Nevertheless, further investigations are required in order to elucidate how gGT induces DC tolerance. In addition, gGT contributes to gastric inflammation via generation of H_2_O_2_, subsequent activation of NF-*κ*B, and upregulation of IL-8 in primary gastric epithelial cells [70]. In a more recent report Rimbara et al. propose glutamine deprivation induced by gGT to be responsible for induction of gastric inflammation and to increase the risk of developing gastric cancer [71].

### 3.4. dupA

dupA is an interesting and as yet not fully characterized *H. pylori* virulence factor involved in inflammation. An association between dupA and increased expression levels of IL-8 has been observed in the gastric mucosa of *H. pylori*-infected subjects [72–74], but neither dupA1 nor dupA2 were found to induce IL-8 secretion by gastric epithelial cells. dupA1 was found, however, to increase proinflammatory cytokine expression, most markedly IL-12p40, IL-12p70, and IL-23 by CD14+ mononuclear cells, which may explain how dupA1 contributes to gastric inflammation [73].

## 4. Metabolism of *H. pylori*

In addition to potential virulence factors and adhesion molecules with a direct effect on host cells, mostly explained above, there are some further metabolic mechanisms that are not *per set* considered as virulence factors. These must be taken into account as potential therapeutic or prophylactic targets in the context of chronic colonization of human stomach. *H. pylori* is a microaerophilic organism that requires a small amount of oxygen (3 to 7 percent) for its metabolic activities and cannot be grown at higher oxygen concentrations like fully aerobic microorganisms [75]. Through whole genome sequencing of *H. pylori* in experimental studies of the bacterial metabolism, it has been inferred that several pathways are missing for the biosynthesis of essential amino acids, lipids, and nucleotides in comparison to other microorganisms like *E. coli*. Whilst amino acids and lipids can also be potential sources of carbon and energy [76, 77], glucose appears to be the only source of carbohydrate utilized by the bacterium [78]. It has been reported that *H. pylori* exploits not only oxidative phosphorylation but also fermentation processes [79]. *H. pylori*, like other living organisms, requires metal ions, specifically cobalt, iron, and nickel, mostly for activity or synthesis of its enzymes [80–82]. Furthermore, *H. pylori* infection may cause metabolic disorders of the host, such as iron deficiency anemia, due to either direct absorption of these trace elements by bacterium or by hampering their uptake or trafficking [83–85].

At the time of *H. pylori*'s discovery by Marshall and Warren, it was reported that this bacterium did not possess the fermentative mechanism and was unable to catalyze carbohydrates [86]. Just a few years later Mendz and Hazell discovered enzymes of the pentose phosphate pathway as well as glucokinase, which were the first suggestions that *H. pylori* had the ability to utilize glucose [87]. Phosphorylated glucose is processed through the pentose phosphate pathway. Its metabolite ribose 5-phosphate is essential for DNA synthesis and repairs [88]. Alternatively, glucose 6-phosphate enters the Entner-Doudoroff pathway and results in pyruvate production [89]. The fate of pyruvate in *H. pylori* has been the subject of several studies [79, 90, 91]. It may be metabolized to acetyl coenzyme-A (acetyl-CoA) and enter the krebs' cycle to produce succinate or fatty acid synthesis, or it may pass the fermentation and lead to the production of acetate, ethanol, fumarate, and lactate [57, 82–89, 89–94]. While some enzymes involved in these metabolic pathways like fumarate reductase are described as potential targets for vaccine development, some downstream metabolites like acetaldehyde (produced by aldehyde—and alcohol dehydrogenase) are known virulence factors.

Amino acids are considered as the main source of nitrogen and to a lesser extent potential carbon and energy reserves of the bacterium. More simply, when glucose or the metabolic enzymes involved in its pathways are lacking, *H. pylori* is able to catalyze amino acids such as arginine, aspartate, asparagine glutamine, and serine and use them as basic nutrients [76, 90]. Some surprisingly novel insights regarding enzymes as well as the metabolites involved in amino acid metabolism have been uncovered in subsequent investigations after descriptions of amino acid requirements [76, 95] and their metabolism. Certain unique properties result in some of these, like *γ*-glutamyltranspeptidase, catalase, high temperature requirement A (HtrA), and fumarate reductase being described as virulence factors and they have been considered as potential candidates for therapeutic as well as prophylactic approaches against *H. pylori* [67, 94, 96–98]. In addition to amino acids, *H. pylori* is able to utilize other substrates like urea and ammonia as source of nitrogen [4, 76]. Amino nitrogen is essential for the synthesis of other biomolecules, and early studies have demonstrated that urea derived nitrogen is incorporated into amino acids [99, 100]. Out of many extensive investigations regarding urea and urease, it is important to point out that the presence of large amounts of urease in the cytoplasm and also in the extracellular milieu of *H. pylori *is unique [101]. Urease is constitutively expressed by *H. pylori* and comprises more than 10% of the whole protein content produced by *H. pylori* [102]. This highly active enzyme is the main responsible factor for the production of ammonia, which beside involvement in biosynthesis also acts in acid resistance [102]. It has been clearly shown that urease is a critical virulence factor essential for the colonization of stomach. These specific properties have designated an exclusive position for urease in vaccine research [103, 104] and must inform successful diagnostic approaches for *H. pylori* [105–107]. It should be stressed that in this paper only a few metabolic mechanisms connected to the virulence of *H. pylori* are mentioned. Other reviews provide a comprehensive description of the *H. pylori*'s metabolism [2, 87, 88].

## 5. Symbiotic Relation between *H. pylori* and Human

The prevalence of *H. pylori* infection is higher in developing countries than that in developed countries. There is evidence that while prevalence of *H. pylori* infection is decreasing in many countries due to improvements in sanitation and living conditions, the prevalence of allergic diseases like asthma and rhinitis has increased by 32% in Western populations [108, 109]. Such a dramatic increase within a relatively short period of time cannot be attributed to genetic determinants alone. Hence, environmental factors are thought to act as major risk factors for the development of asthma. The inverse relationship between infectious and atopic diseases in Western countries has paralleled decreasing rates of serious infections due to both increased hygiene standards and the expanded availability of antibiotics. This relationship between infection and allergic diseases has led to the formation of the hygiene hypothesis [110]. More recent studies attribute this relationship to a shift of balance between effector T cell subtypes towards Th2-helper cells in the absence of early exposure towards pathogens [111, 112]. Taken together, these observations suggest that the observed increase in the prevalence of asthma may be linked to a decrease in infections, while certain pathogens like respiratory viruses may actually enhance the development of asthma [113, 114]. Although the current data mostly relies on epidemiological associations, a few functional or mechanistic links have been established [111, 115]. In this context, several recent studies have investigated the association of *H. pylori* infection and allergic disease, and increasing data are indicative of an inverse association of *H. pylori* with asthma and allergy [116, 117]. The acquisition of *H. pylori* in childhood seems to be linked to reduced asthma and allergy risk [118]. Recently, a huge cross-sectional analysis, using data from 7412 participants in the National Health and Nutrition Examination Survey (NHANES), revealed that *H. pylori* seropositivity was inversely associated with onset of asthma before 5 years of age and current asthma in children aged 3–13 years [119]. Despite the strong statistical power of the study, these results are still intensively debated [120, 121]. This is perhaps to be expected given the socioeconomic impact of both diseases. It is important to point out that strategies aiming at broadly eradicating *H. pylori* in order to prevent gastric cancer might have unexpected consequences on asthma prevalence. Therefore, not only is a multilateral knowledge of *H. pylori* as a complex pathogen necessary, but also, as Martin Blaser states, “*Prospective studies are needed to understand causal relationships and to help ascertain intermediate mechanisms*” [122].

## 6. Conclusion

A better understanding of “multidirectional” aspects and features of *H. pylori*'s biology is of fundamental interest to develop strategies helping us to cope with this infection. This minireview attempts to emphasize some of these different features. While the importance and impact of *H. pylori*'s biochemical virulence factors on host physiology are not negligible, adhesion molecules and mechanisms by which the bacterium can anchor and nestle in the human stomach are similarly meaningful. Additionally a comprehensive knowledge of the unique metabolism of the bacterium will help in identifying possible foibles which may be applicable for future therapies. The complex combination of environmental, host, and bacterial factors determines the susceptibility and severity of outcome of *H. pylori* infection and related pathology in the subset of individuals. Important epidemiological as well as new experimental findings have confirmed the validity of the “*Hygiene hypothesis*” also in relation with *H. pylori*. These data and future studies revealing the distinct beneficial mechanism of *H. pylori*'s contribution to the “*Hygiene hypothesis*” will guide us in developing new drugs for allergic and immune relevant applications. Additionally, new diagnostic tests suitable for screening of larger populations will facilitate to establish risk adjusted guidelines of *H. pylori* control.
